# Interleukin-17 receptor A drives cancer stem-like properties in colorectal cancer through STAT3 activation

**DOI:** 10.7150/jca.121654

**Published:** 2026-01-01

**Authors:** Jeng-Kai Jiang, Chi-Hung Lin, Chun-Chi Lin, Liang-Chuan Lo, Po-Yen Sung, Zhen-Yu Wen, Chien-Ping Lin, Ting-An Chang, Chih-Yung Yang

**Affiliations:** 1 School of Medicine, National Yang Ming Chiao Tung University, Taipei, Taiwan.; 2 Division of Colon & Rectal Surgery, Department of Surgery, Taipei Veterans General Hospital, Taipei 112304, Taiwan.; 3 Division of Colon & Rectal Surgery, Department of Surgery, West Garden Hospital, Taipei 108035, Taiwan.; 4 Institute of Microbiology and Immunology, National Yang Ming Chiao Tung University, Taipei 112304, Taiwan.; 5 Department of Biological Science and Technology, National Yang Ming Chiao Tung University, Hsinchu 30093, Taiwan.; 6 National Genomics Center for Clinical and Biotechnological Applications, Cancer and Immunology Research Center, National Yang Ming Chiao Tung University, Taipei 112304, Taiwan.; 7 Cancer and Immunology Research Center, National Yang Ming Chiao Tung University, Taipei 112304, Taiwan.; 8 Department of Pathology, Ren-Ai Branch, Taipei City Hospital, Taipei 10629, Taiwan.; 9 General Education Center, University of Taipei, Taipei 100234, Taiwan.; 10 Department of Education and Research, Taipei City Hospital, Taipei 10629, Taiwan.

**Keywords:** interleukin-17 receptor A, cancer stem cell, self-renewal, STAT3, colorectal cancer

## Abstract

Cancer stem cells (CSCs) play pivotal roles in tumor relapse, metastasis, and therapy resistance. Interleukin 17 receptor A (IL-17RA) is a key mediator in colorectal cancer (CRC) pathogenesis and progression. Our recent study demonstrated that reduced IL-17RA expression correlates with favorable prognosis in CRC patients and suppresses tumor growth in murine models. This study aimed to investigate the role of IL-17RA in promoting cancer stem-like properties and its impact on colorectal cancer prognosis and chemoresistance. Expression levels of IL-17RA and CSC markers in CRC cells were evaluated using quantitative real-time polymerase chain reaction and Western blotting. Kaplan-Meier analysis of 68 CRC patients revealed that high IL-17RA expression is associated with poor clinical outcomes. To investigate IL-17RA's functional role, CRC cells with stable IL-17RA overexpression were analyzed for changes in CSC marker expression, sphere formation, and 5-fluorouracil (5-FU) resistance. IL-17RA overexpression significantly increased CSC marker expression, including cluster of differentiation 133 (CD133), leucine-rich repeat-containing G protein-coupled receptor 5 (LGR5), sex determining region Y-box 2 (SOX2), and enhanced tumor sphere formation and 5-FU resistance in SW620 cells. Specific inhibitors of IL-17RA signaling, such as the STAT3 inhibitor Stattic, reduced the expression of CD133, LGR5, ALDHA1, SOX2 and c-MYC, as well as tumor sphere formation in SW620 cells. These findings elucidate a novel IL-17RA-STAT3 axis that regulates CSC properties in CRC and highlight IL-17RA as a promising prognostic biomarker and therapeutic target for CRC treatment.

## 1. Introduction

Colorectal cancer (CRC) is among the most prevalent malignancies, ranking third in incidence and second in mortality worldwide in recent years 1. According to the American Cancer Society statistics, 5-year survival drops to 14% with the spread of tumor to distant organs, also known as advanced cancer disease 2. The precise prognosis prediction can guide disease monitoring strategies and improve patient survival rate 3. Drug resistance and molecular heterogeneity are the primary causes of high mortality in metastatic cancer. Clinically, approximately 20% of the patients present with metastases at diagnosis 4.

A subset of cancer stem cells (CSCs) within the bulk tumor are significantly more tumorigenic than other cells, contributing to drug resistance and driving tumor initiation and progression due to their capacity for self-renewal, differentiation into diverse cancer cell lineages, and the expression of stem cell markers shared with normal stem cells 5, 6. The transmembrane glycoproteins cluster of differentiation 133 (CD133), aldehyde dehydrogenase 1 (ALDH1), and leucine-rich repeat-containing G protein-coupled receptor 5 (LGR5) are CSC markers in CRC 7, 8 and other various cancers 9-11. Other transcription factors known as CSCs markers in CRC are sex determining region Y-box 2 (SOX2), homeobox protein NANOG (NANOG), octamer-binding transcription factor 4 (OCT4), and c-MYC, which govern the differentiation and self-renewal of pluripotent stem cells 12-14.

Interleukin 17 (IL-17), a highly potent inflammatory cytokine, plays a significant role in autoimmune disorders like rheumatoid arthritis and multiple sclerosis 15 and is linked to the promotion of cancer progression in CRC and other various tumor types 16, 17. Furthermore, IL-17 binds to IL-17 receptors A (IL-17RA), subsequently triggering the activation of MAPKs, NF-κB, C/EBP and JAK2/STAT3 signaling pathways 18, 19. The IL-17/IL-17RA pathway mediates this cascade via a downstream positive circuit, directly stimulating tumor formation 20. *In vivo*, the IL-17A pathway suppression has been associated with decreased tumor growth 21-24, increased tumor cell apoptosis 21, 22, decreased metastasis 23, 25, and increased survival 23, 25. In contrast, stimulation of the IL-17-IL-17 receptor signaling pathway leads to the opposite effect 26, 27. The role of IL-17RA engagement in promoting tumor development and progression has been identified in gastric cancer, non-small cell lung cancer, and osteosarcoma 28-30. In our previous studies, changes in serum IL17 levels correlated with the dynamics of circulating tumor cells. IL-17 expression was significantly higher in patients with CRC with recurrence than in those without recurrence 31. Additionally, decreased expression of interleukin-17RA in patients with CRC correlates with better prognosis and inhibits tumor growth and vascularity in murine models 32. Implication of IL-17RA in the renewal of glioma cells suggested its potential role in promoting CSC and tumor advancement 33.

In this study, we evaluated the role of IL-17RA in CSC and the signaling mechanisms that affect CSC properties. We found that high IL-17RA expression in patients with CRC tumors was negatively correlated with survival and positively correlated with the expression of CSC markers CD133, LGR5, and ALDH2, and CSC markers SOX2 and c-MYC. IL-17RA stable overexpression cells were used to examine changes in CSC markers, sphere formation, and the regulatory mechanisms of the signaling pathway. Specific IL-17RA signaling inhibitors evaluated the effects of IL-17RA signaling on the expression of CSC markers and sphere formation capability.

## 2. Materials and Methods

### 2.1 CRC patients and sample collection

In this retrospective study, 68 CRC tissue samples were obtained from the Biobank of Taipei City Hospital (Taipei, Taiwan) for analysis between January and December 2019. The tissues were originally collected from patients who underwent surgical resection between January 2012 and December 2014. Before the time of surgery, all patients provided written informed consent agreeing to donate their tissue specimens for long-term storage in the Biobank and for future research use. All patients received standard clinical treatment according to institutional protocols in place at the time. The present study was approved by the Research Ethics Committee of Taipei City Hospital, Taipei, Taiwan, on October 5, 2018 (TCHIRB number: 10709102-E). The age range of the patients was 35 to 89 years. Histological examination determined the staging of primary tumors. Table 1 shows the demographic and clinicopathological characteristics of patients with CRC.

### 2.2 Cell lines and culture

The SW480 (primary colon adenocarcinoma) and SW620 (subsequent lymph node metastasis) cell lines are derived from the same patient. Two isogenic human colon cancer cell lines, SW480 (RRID: CVCL_0546) derived from the primary tumor and SW620 (RRID: CVCL_0547) from lymph node metastasis, were sourced from the Bioresource Collection and Research Center (BCRC, Hsinchu, Taiwan) 34. These cells were cultured in DMEM (Gibco) supplemented with 10% FBS (Gibco), 100 IU/ml penicillin, and 100 μg/ml streptomycin. All culture reagents were purchased from Invitrogen (Carlsbad, CA, USA). All cell lines were authenticated using short tandem repeat (STR) profiling within the last 3 years by Topgen Biotechnology (Kaohsiung City, Taiwan). All experiments were performed with mycoplasma-free cells. Plasmid constructs, maintained in a humidified atmosphere at 37 °C with 5% CO2, expressing the human IL-17RA gene were employed to establish stable IL-17RA overexpression cell lines of SW480 #1 and #2 and SW620 #3 and #4 via lentiviral transduction. The human IL-17RA gene ORF cDNA clone, sourced from Sino Biological Inc. (Catalog Number: HG10895-G), was sequenced to confirm the correct human IL-17RA ORF sequence. Transduced cells were cultured in DMEM (Gibco) supplemented with 20 μg/mL blasticidin (A1113903; Thermo Fisher Scientific).

### 2.3 Lentivirus production and transduction

293T cells (RRID: CVCL_0063) were obtained from the American Type Culture Collection (ATCC, Manassas, VA, USA) and cultured in DMEM (Gibco) supplemented with 10% FBS (Gibco) under 37°C in a humidified atmosphere of 5% CO₂. Cells were seeded on 6-well plates (1 × 10⁶ cells/well) to produce lentiviruses for SW480 and SW620 infection. On next day, 293T were transfected with 2 μg of lentiviral vectorspLAS3w.Pbsd (National RNAi Core Facility, Acadamic Sinica, Taiwan) containing hIL-17RA ORF, 0.9 μg of pCMV-deltaR8.91(National RNAi Core Facility, Acadamic Sinica, Taiwan), and 0.1 μg of pMD.G (National RNAi Core Facility, Acadamic Sinica, Taiwan) with T-Pro NTR II transfection reagent (T-Pro Biotechnology, Taiwan). After 24 h, the medium was replaced with DMEM (Gibco) supplemented with 30% FBS (Gibco). After 48 h, supernatants were harvested. Cell lines were seeded in a 6-well plate (1× 10^6^ cells per well) with the feeder-free system for infection and subsequently treated with virus [multiplicity of infection (MOI) = 10] for 24 h. On the next day, 20 μg/mL blasticidin (A1113903; Thermo Fisher Scientific) selected the cells.

### 2.4 Quantitative real-time RT-PCR

Tissue samples were processed with a homogenizer for effective disruption prior to RNA extraction. Total RNA was extracted using TRIzol Reagent (Life Technologies) following the manufacturer's protocol. Reverse transcription was carried out using the RevertAid First Strand cDNA Synthesis Kit (Thermo Fisher Scientific). Gene expression was quantified by real-time quantitative PCR with the QuantiFast SYBR Green PCR Kit (QIAGEN) on a 7900HT Fast Real-Time PCR System (Applied Biosystems). Relative expression levels were calculated using the 2^-ΔΔCT^ method, as previously described by Livak and Schmittgen 35. Initial heat activation: 95 °C for 5 minutes, 40 cycles: Denaturation: 90 °C for 10 seconds; Annealing: 60°C for 30 seconds. GAPDH was used as the internal control for normalization in RT-qPCR. The following primer sequences were used: IL-17RA, 5'-ATGGACACTGCAGACAGACG-3' (forward) and 5'-CTCACAGTCAGGCACAAGGA-3' (reverse); CD133, 5'- TTCTTGACCGACTGAGACCCA-3' (forward) and 5'- TCATGTTCTCCAACGCCTCTT-3' (reverse); LGR5, 5'- GGTCGCTCTCATCTTGCTCA-3' (forward) and 5'- GCCACAGGGCAGTTTAGGAT-3' (reverse); ALDH1, 5'- AGCCTTCACAGGATCAACAGA-3' (forward) and 5'- GTCGGCATCAGCTAACACAA-3' (reverse); SOX2, 5'- GCCGAGTGGAAACTTTTGTCG-3' (forward) and 5'-GGCAGCGTGTACTTATCCTTCT-3' (reverse); c-MYC, 5'- CACCAGCAGCGACTCTGA-3' (forward) and 5'- GATCCAGACTCTGACCTTTTGC-3' (reverse); GAPDH, 5'- TGGTATCGTGGAAGGACTCATGAC-3' (forward) and 5'- ATGCCAGTGAGCTTCCCGTTCAGC-3' (reverse). Primers targeting CD133 were designed based on the PROM1 gene sequence (which encodes the CD133 protein), as PROM1 and CD133 are used interchangeably in the literature 36, 37. Primer specificity was verified using NCBI BLAST (https://blast.ncbi.nlm.nih.gov/).

### 2.5 Western blot

Cells were lysed using RIPA solution supplemented with cOmplete™ Protease Inhibitor Cocktail (Roche). Proteins were then separated by sodium dodecyl sulfate-polyacrylamide gel electrophoresis and transferred to PVDF membranes (EMD Millipore, Billerica, MA, USA). Following a 1-hour blocking step at room temperature with 5% bovine serum albumin (BSA) in Tris-buffered saline with 0.1% Tween 20 (TBS-T), the membranes were incubated with primary antibodies at 4°C overnight: anti-IL-17RA (Cell Signaling Technology, Cat. No: #12661, dilution 1: 1000), anti-CD133 (Cell Signaling Technology, Cat. No: #5860, dilution 1: 1000), anti-LGR5 (GeneTex, Cat. No: GTX129862, dilution 1: 1000), anti-ALDH1 (GeneTex, Cat. No: GTX123973, dilution 1: 1000), anti-SOX2 (GeneTex, Cat. No: GTX101507, dilution 1: 1000), anti-cMYC (GeneTex, Cat. No: GTX103436, dilution 1: 1000), anti-phospho-STAT3 (Ser727) (Cell Signaling Technology, Cat. No: #9134, dilution 1: 1000), anti-STAT3 (Cell Signaling Technology, Cat. No: #12640, dilution 1: 1000), anti-phospho-p38 (Cell Signaling Technology, Cat. No: #4511, dilution 1: 1000), anti-p38 (Cell Signaling Technology, Cat. No: #8690, dilution 1: 1000), anti-β-CATENIN (Cell Signaling Technology, Cat. No: #8480, dilution 1: 1000), anti-TUBULIN (GeneTex, Cat. No: GTX101279, dilution 1: 3000) and anti-GAPDH (GeneTex, Cat. No: GTX100118 dilution 1: 3000). The membranes were washed three times for 10 minutes each with TBS-T and then incubated for 1 hour at room temperature with horseradish peroxidase-conjugated secondary antibodies (Jackson ImmunoResearch, Cat. No: 711-035-152, dilution 1: 5000). Western blot signals were visualized using SuperSignal West Femto Maximum Sensitivity Substrate (Thermo Fisher Scientific) and captured with an ImageQuant LAS-4000 (GE Healthcare Life Sciences). GAPDH was used as a loading control for normalization in Figure 5, and β-TUBULIN was used as a loading control for normalization in Supplementary Figure 3A and 3C. 38 For Figure 5A, phosphorylated STAT3 (p-STAT3) was first detected. The membrane was then stripped using a mild stripping buffer (25 mM glycine, pH 2.0, 1% SDS) at room temperature for 10-15 minutes, followed by thorough washing. The same membrane was subsequently reprobed for total STAT3 and the internal control GAPDH to ensure consistent loading and signal comparison.

### 2.6 Tumor sphere formation assay

Cells (SW620 and SW480) were seeded into ultra-low attachment 96-well plates (Corning, New York, USA) at a density of 2 × 10² cells per well and cultured in DMEM (Gibco) supplemented with 20 ng/ml bFGF (Sigma-Aldrich, St. Louis, Missouri, USA), 20 ng/ml human recombinant EGF (Sigma-Aldrich, St. Louis, Missouri, USA), and B27 (1:50, Invitrogen) for 7 days. The tumor sphere cells larger than 500 μm^2^ in area were counted on the 4 fields of view per well using Image J under an inverted microscope at 100X magnification. The average number and size of the spherical cells were calculated and plotted.

### 2.7 *In vitro* cytotoxicity assay

The cytotoxicity of 5-FU was assessed in all mentioned cell lines at concentrations of 0, 1, 10, 25, 50, 100, 200, and 300 µM. After 72 hours of incubation with 5-FU, the culture medium was replaced with fresh medium containing 10% (v/v) MTT solution (0.5 mg/mL). Following 2 hours of incubation, the medium was removed, and the formed formazan crystals were dissolved in 100 µL of DMSO. Absorbance was measured at 550 nm using a microplate spectrophotometer (Thermo Fisher Scientific). Three independent experiments with three replicates each were performed. The IC50, defined as the concentration that inhibited 50% of cellular metabolic activity, was determined using nonlinear regression (four-parameter logistic model) in GraphPad Prism (v6.0) software. Results are presented as the mean ± standard deviation (SD).

### 2.8 Statistical analyses

All analyses were performed using GraphPad Prism (v6.0). and data are expressed as the mean ± SD of at least three independent experiments. Differences with p < 0.05 were considered statistically significant. Continuous variables were reported as median values with ranges. Survival analysis was performed using the Kaplan-Meier method and compared using the log-rank test. ROC curve analysis was also conducted. Normality of the data was assessed using Anderson-Darling test 39. Depending on the outcome, Pearson's correlation was used for normally distributed data, and Spearman's correlation was used for non-normally distributed data. For comparisons among more than two groups, one-way ANOVA followed by Dunnett's test was used. For experiments involving two independent variables, two-way ANOVA followed by Sidak's multiple comparisons test was applied. Unpaired two-tailed t-tests were used only for comparisons between two independent groups. Paired t-tests were not applied, as none of the data were paired. The statistical tests used for each figure are indicated in figure legends.

## 3. Results

### 3.1 IL-17RA expression significantly associated with the survival outcome and positively correlates with stemness markers in CRC tumors

**Figure 1:**
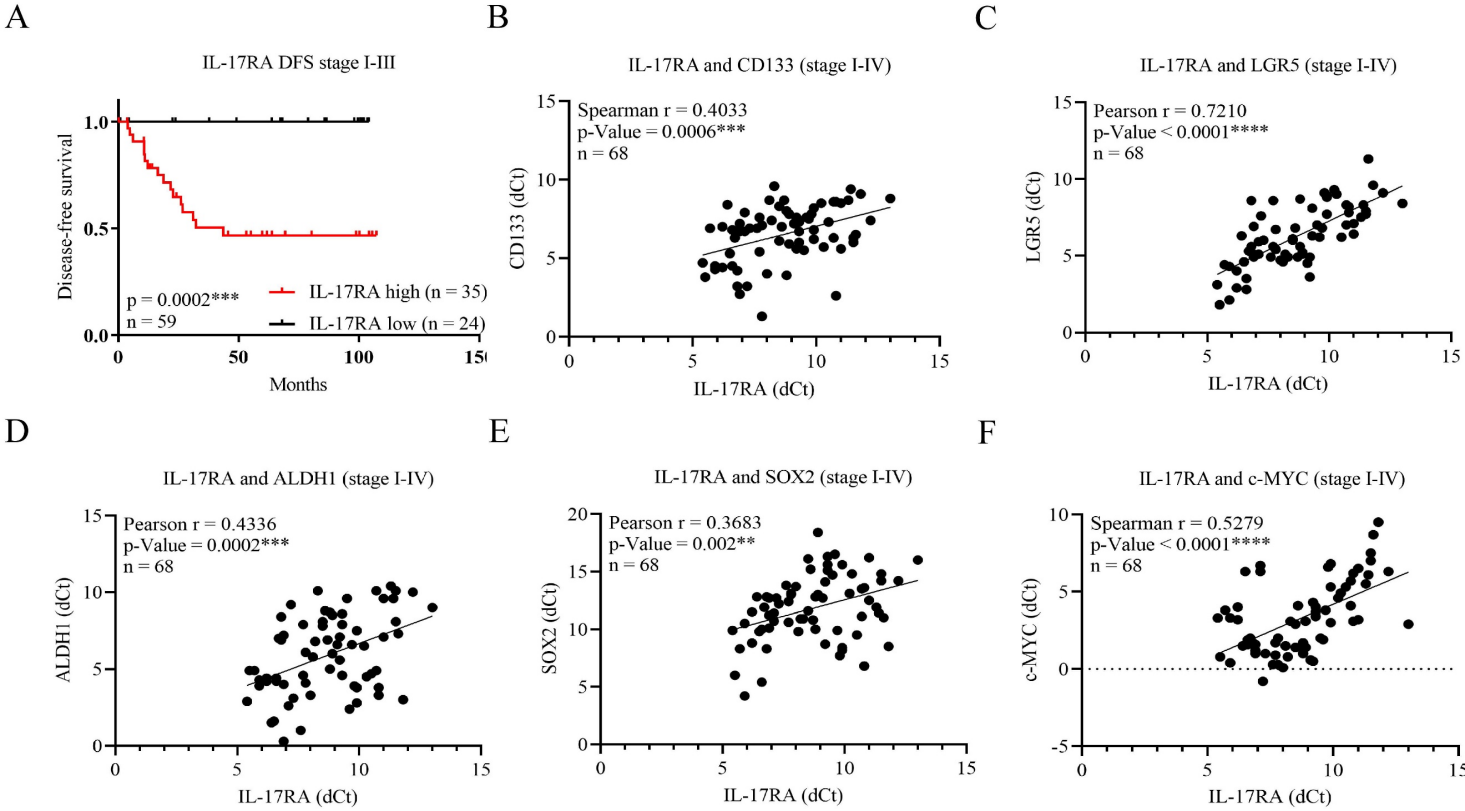
Overexpression of interleukin-17 receptor A (IL-17RA) in colorectal cancer tissues is correlated with poor-disease outcome and cancer stem cell marker expression. (A) Kaplan-Meier analysis of disease-free survival according to IL-17RA expression (*p* = 00002, N=59). Pearson analysis showed that IL-17RA expression was positively related with the expression of stem cell markers cluster of differentiation 133 (CD133) (B)(*p*=0.004), leucine-rich repeat-containing G protein-coupled receptor 5 (LGR5) (C)(*p*<0.0001), aldehyde dehydrogenase 1 (ALDH1) (D)(*p*=0.0002) and stemness gene sex determining region Y-box 2 (SOX2) (E) (*p*=0.002) and c-MYC (F)(*p*<0.0001). Correlation analysis was conducted using Spearman's rank correlation coefficient or Pearson's correlation coefficient, as appropriate. *p < 0.05, **p < 0.01, ***p < 0.001.

We examined the expression of IL-17RA in human CRC using qRT-PCR to assess the expression levels of IL-17RA and the prognosis of patients with CRC. Data from 68 patients with CRC were divided into two groups according to the differential expression of IL-17RA (Table 1). Patients with high IL-17RA expression showed significantly worse overall survival (OS) (*p* = 0.0004), disease-free survival (DFS) (*p* = 0.0002), and disease-specific survival (*p* = 0.0004) (Figure 1A and Supplementary Figure 1). The results indicated that cancer with higher expression levels of IL-17RA significantly correlated with elevated mortality rates. Analysis of correlation of the expression of IL-17RA with CSC markers in 68 CRC samples revealed that IL-17RA expression positively correlated with the expression of stem cell markers CD133 (*p* = 0.0006), LGR5 (*p* < 0.0001) and ALDH1 (*p* = 0.0002), and stemness genes SOX2 (*p* = 0.002) and c-MYC (*p* < 0.0001) (Figure 1B-F).

### 3.2 Overexpression of IL-17RA can upregulate the expression of stem cell markers and stemness genes

**Figure 2:**
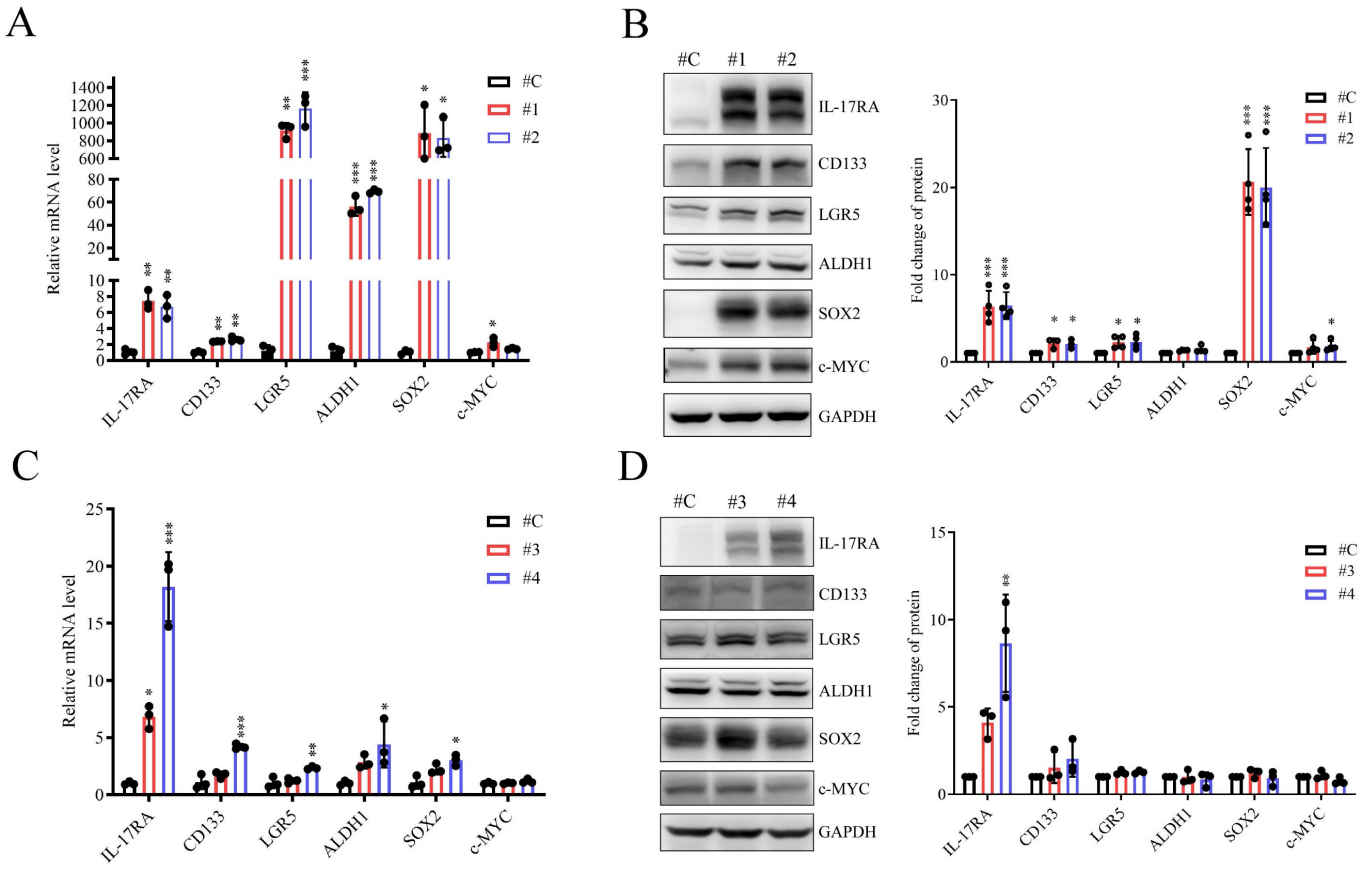
Interleukin-17 receptor A (IL-17RA) overexpression promotes the expression of cancer stem cell markers in SW620 (A, B) and SW480 (C, D) cells. mRNA and protein expression of the genes are detected by quantitative RT-PCR (A, C) and Western blot (B, D) respectively. Data are presented as mean ± SD. Statistical significance was determined using one-way ANOVA followed by Dunnett's test. *p < 0.05, **p < 0.01, ***p < 0.001.

To test the role of IL-17RA as a modulator of CSC marker expression, we generated human SW620 and SW480 CRC cells that stably overexpressed IL-17RA. The RNA and protein levels of IL-17RA-affected CRC markers were examined. The expression of CD133, LGR5, and SOX2 was significantly increased in IL-17RA-overexpressing SW620 cells compared to control cells (Figure 2A and B). In SW480 cells overexpressing IL-17RA, there is an increasing trend in the mRNA levels of CD133, LGR5, ALDH1, and SOX2. However, at the protein level, CD133, ALDH1, and SOX2 show no significant difference, while LGR5 exhibits a slight increasing trend. (Figure 2C and D). IL-17RA overexpression significantly downregulated CD44 expression but did not affect OCT4 or NANOG expression in SW620 and SW480 cells (Supplementary Figure 2). These findings suggested that IL-17RA overexpression enhances the expression of the specified CSC markers.

### 3.3 Overexpression of IL-17RA cells possess self-renewal activity

**Figure 3:**
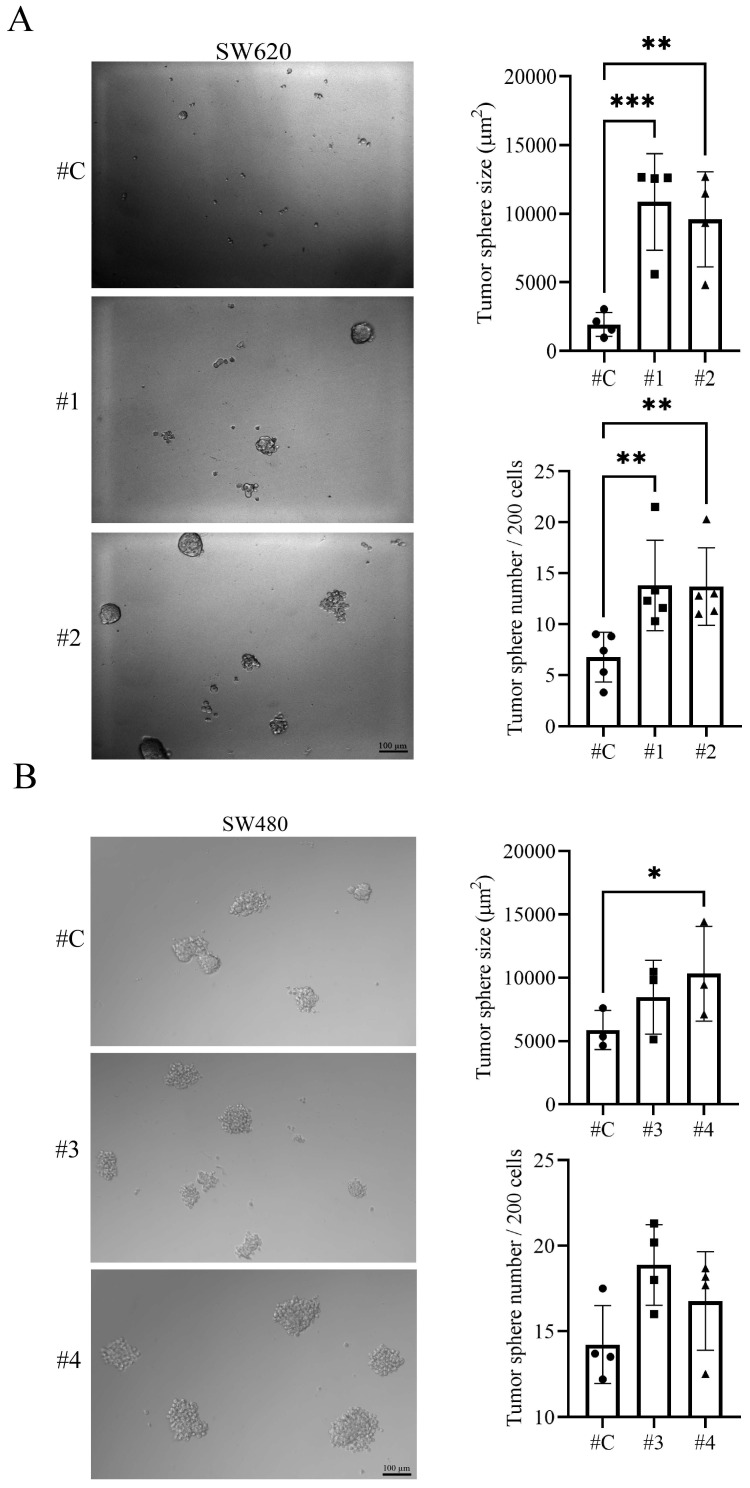
Interleukin-17 receptor A (IL-17RA) promotes sphere formation capability. Sphere-forming assay of IL-17RA overexpressing SW620 (A) and SW480 (B) cells is assessed. Data are presented as mean ± SD. Statistical significance was determined using one-way ANOVA followed by Dunnett's test. *p < 0.05, **p < 0.01.

An *in vitro* model to investigate the IL-17RA-contributed property of CSCs in CRC is required. The sphere formation assay is a widely used *in vitro* technique for identifying and studying the properties of CSCs. The SW480 control cells showed better sphere formation size and number than the SW620 control cells after 7 days. SW620 cells with IL-17RA stable expression were cultured in a spherical medium for 7 days. The number and size of sphere in IL-17RA- overexpressing SW620 cells were significantly higher than those in SW620 control cells (Figure 3A). In addition, the number and size of sphere in IL-17RA-overexpressing SW480 cells showed an increasing trend compared with those in SW480 control cells (Figure 3B). Our results showed that IL-17RA overexpression enhanced sphere formation and self-renewal.

### 3.4 Overexpression of IL-17RA Promotes Drug Resistance Capability

**Figure 4:**
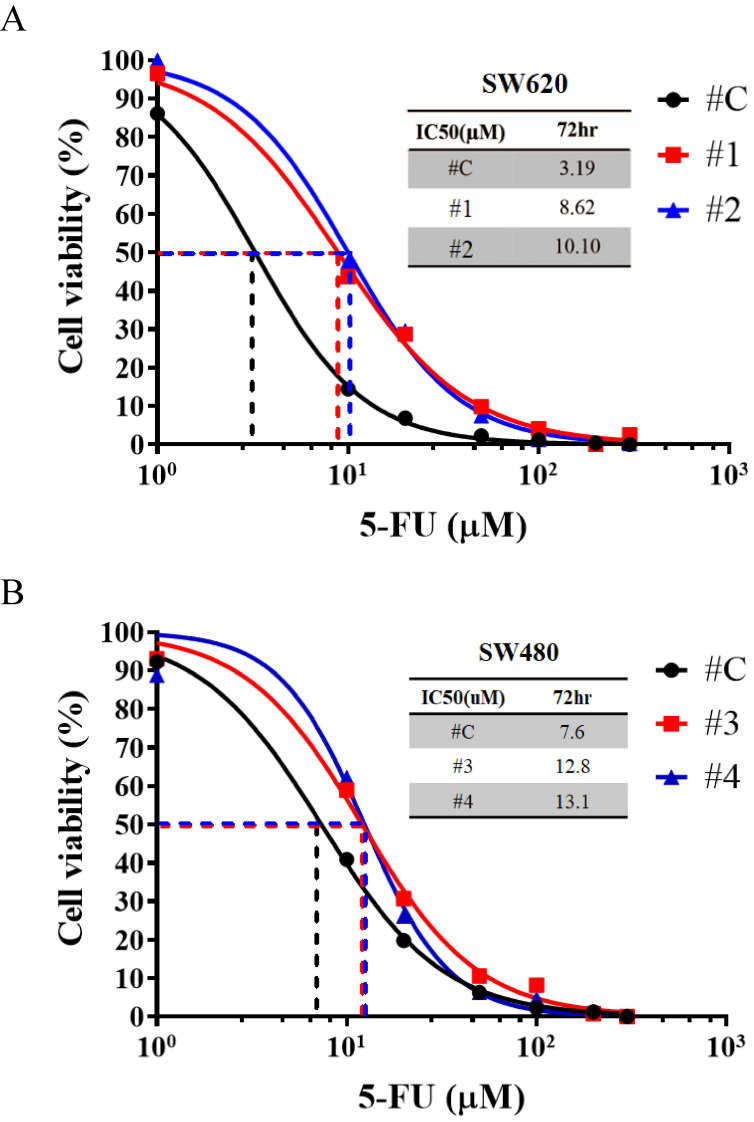
The 5-flurouracil (5-FU) cell toxicity test and half maximal inhibitory concentration (IC50) concentration measurement in IL-17RA-overexpressed SW620 (A) and SW480 (B).

Chemotherapeutic resistance of CSCs is responsible for cancer recurrence and metastasis 40, 41. We tested the cytotoxic effects of 5-flurouracil (5-FU) on CRC cells overexpressing IL-17RA because 5-FU is one of the commonly used chemotherapeutic drugs for treating CRC. Compared with control cells, the IC50 value of 5-FU significantly increased upon IL-17RA overexpression in SW620 cells (Figure 4A). In the other cell SW480, IC50 value significantly elevated with IL-17RA overexpression (Figure 4B). The IL-17RA overexpressed SW480 cells were less sensitive to 5-FU treatment than IL-17RA overexpressed SW620 cells and showed significant difference compared with control cells in the MTT assay and IC50 concentration. These results indicated that elevated IL-17RA expression could enhance 5-FU resistance in SW620 and SW480 cells.

### 3.5 IL-17RA overexpression promotes cancer stem-like properties of colorectal cancer cells via STAT3 activation

**Figure 5:**
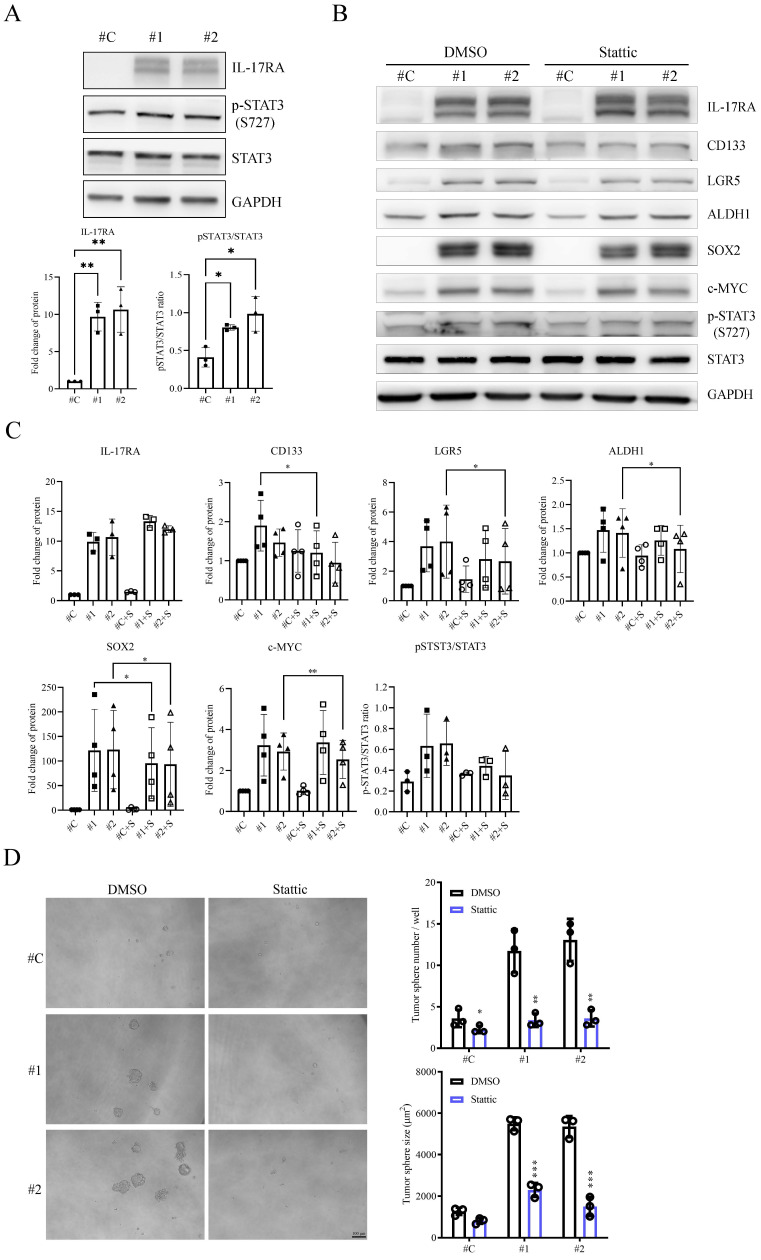
The signal transducer and activator of transcription 3 (STAT3) inhibitor Stattic can suppress the cancer stem cell (CSC) proteins expression in IL-17RA overexpressed SW620 cells. (A) IL-17RA overexpression can activate the STAT3 phosphorylation. (B) Stattic inhibited the indicated CSC markers. (C) Quantification of Western blot results in (B), based on three independent experiments. (D) Sttatic can significantly suppress the ability of sphere-formation. Data are presented as mean ± SD. Statistical significance was determined using one-way ANOVA followed by Dunnett's test for (A) and two-way ANOVA followed by Sidak's multiple comparisons test for (C). Statistical significance was determined using a multiple-group unpaired t-test for (D). *p < 0.05, **p < 0.01, ***p < 0.001.

We treated IL-17RA-overexpressing SW620 cells with various pathway inhibitors to investigate the IL-17RA-triggered signal transduction pathway that contributes to CSC properties (Figure 5). Compared with control SW620 cells, the WNT pathway inhibitor XAV939 and the MAPK inhibitor SB203580 did not affect the expression of stem cell and stemness markers in IL-17RA-overexpressing SW620 cells (Supplementary Figure 3). However, the STAT3 inhibitor, Stattic, significantly decreased SOX2 expression and showed a decreasing trend in the expression of CD133, LGR5, c-MYC, and ALDH1A1 in IL-17RA-overexpressing SW620 cells. (Figure 5B and C). Overexpression of IL-17RA activated STAT3 phosphorylation in SW620 cells. Stattic effectively suppressed STAT3 phosphorylation and reduced both the number and size of tumor spheres formed (Figure 5A and D). Additionally, Stattic exhibited significant cytotoxic effects and enhanced the cytotoxicity of 5-FU in both control SW620 cells and IL-17RA-overexpressing SW620 cells (Supplementary Figure 4).

## 4. Discussion

Previous studies have established that IL-17RA plays a role in promoting tumor progression, inflammation, and immune evasion in various cancers, including colorectal cancer. For example, IL-17RA has been shown to mediate pro-inflammatory signaling pathways that contribute to tumor growth and resistance to therapy. However, the specific mechanisms by which IL-17RA contributes to cancer stemness remain poorly understood. Our study provides novel evidence that IL-17RA overexpression directly enhances the expression of CSC markers and sphere-forming capacity in colorectal cancer cells. Importantly, we identify STAT3 as a critical downstream effector of IL-17RA signaling that drives the CSC phenotype. Although previous research has implicated STAT3 in the regulation of stemness and IL-17 signaling in cancer, the direct mechanistic connection between IL-17RA and STAT3-mediated CSC properties had not been demonstrated. Thus, our findings reveal a previously unrecognized IL-17RA-STAT3 axis in the regulation of colorectal cancer stemness and suggest new potential therapeutic strategies targeting this pathway.

Although many studies have highlighted that IL-17 and its downstream signaling pathways contribute to poor prognosis in various cancers, including osteosarcoma, NSCLC, gastric cancer, pancreatic carcinoma, and CRC, the impact of IL-17RA on CRC prognosis and progression remains debated. Our recent study found that elevated IL-17RA expression levels are linked to poor outcomes in CRC patients. Reduced IL-17RA expression impairs cellular proliferation, migration, invasion, and EMT gene expression. Additionally, IL-17RA knockdown inhibits tumor vascularity and growth and decreases the population of MDSCs and Tregs in mouse tumors 32.

In this retrospective study, patients with CRC with high IL-17RA expression in their tumor tissue had a poorer prognosis than those with low IL-17RA expression. The expression of CSC markers, including CD133, LGR5, and ALDH1, and stemness genes, such as SOX2 and c-MYC, was significantly and positively correlated with IL-17RA expression in CRC tissues (Figure 1). These results suggest that IL-17RA overexpression may modulate CSC gene expression, contributing to poor prognosis in patients with CRC. A limitation of our study is the lack of normal adjacent tissues as a control group. Our analysis was restricted to comparisons between tumor tissues, which may limit the interpretation of gene expression changes in the broader context of tumor versus normal tissue differences.

The SW620 and SW480 cell lines, derived from different stages of disease progression in the same patient, provide a useful model for studying metastasis and drug resistance in colorectal cancer. We established stable IL-17RA overexpression in SW620 and SW480 cells to evaluate the role of IL-17RA in CSC properties. Overexpression of IL-17RA enhanced the expression of CSC markers in these cells (Figure 2). SW480 control cells have better sphere formation efficiency and 5-FU resistance than SW620 control cells 42, 43. IL-17RA overexpression in SW620 and SW480 cells increased sphere formation efficiency and 5-FU resistance (Figure 3 and 4).

We used inhibitors on IL-17RA-overexpressing SW620 cells to investigate the role of IL-17RA-triggered signal transduction pathways on CSC properties. While WNT and MAPK inhibitors had no effect, the STAT3 inhibitor Stattic significantly decreased the expression of stem cell markers and reduced tumor sphere formation, enhancing the cytotoxicity of 5-FU without being cytotoxic itself (Figure 5 and Supplementary Figure 4).

Our findings suggest that the observed increase in CSC marker expression, spheroid formation, and STAT3 activation upon IL-17RA overexpression may result from both autocrine and ligand-independent mechanisms. Preliminary data indicate that IL-17RA overexpression upregulates IL-17A expression in colorectal cancer cells, suggesting the presence of an autocrine loop that could enhance receptor signaling (Data not shown). In addition, Luo and colleagues recently reported that IL-17RA can undergo autonomous activation independent of ligand binding, sustaining downstream signaling and promoting disease progression 44. Together, these mechanisms provide a plausible explanation for the enhanced IL-17RA signaling activity in our study even in the absence of exogenous IL-17 stimulation.

CSCs are a subpopulation of cancer cells that possess characteristics similar to normal stem cells, such as the ability to self-renew and differentiate into various cell types found in tumors. The relationship between the CSCs and drug resistance is complex and multifaceted. CSCs can adapt and develop resistance mechanisms, including quiescent nature, efflux pump overexpression, efficient DNA repair, anti-apoptotic pathways, and signaling pathways such as Wnt/β-catenin, Hedgehog, and Notch, allowing them to survive chemotherapy and contribute to relapse and treatment failure 40, 41. In this study, IL-17RA overexpression in SW620 and SW480 cells enhanced 5-FU resistance. Various ATP-binding cassette (ABC) transporters, including ABCB1, ABCB7, and ABCD1, actively pumping out chemotherapeutic drugs, were upregulated in IL-17RA-overexpressing SW620 cells (data not shown). The mechanisms underlying IL-17RA-induced drug resistance require ongoing research and adaptation of therapeutic strategies.

LGR5, ALDH1, CD44, and CD133 are critical CSC markers for tumor growth, metastasis, and therapy resistance. Additionally, transcription factors, such as SOX-2, NANOG, OCT4, and c-MYC, are crucial for the pluripotency and self-renewal of CSCs. CD133 expression in cancer cells is associated with enhanced self-renewal, migration, invasion, and survival under stress conditions. This expression is linked to the co-expression of pluripotency genes, such as SOX2, NANOG, OCT4, and c-MYC, which regulate differentiation and self-renewal in pluripotent stem cells 45. LGR5, a receptor involved in the Wnt signaling pathway, is associated with CSC maintenance and aggressive tumor behavior is linked to tumorigenic potential and poor prognosis in cancers 46. ALDH1, an enzyme involved in cellular differentiation, marks CSCs in various cancers, with high activity correlating to self-renewal and chemotherapy resistance 47.

SOX2, a master regulator of embryonic and induced pluripotent stem cells, promotes self-renewal and drug resistance, fuels tumor initiation, and contributes to tumor aggressiveness in CRC 14. c-MYC, a key regulator of cell growth and proliferation, is frequently deregulated in cancers and contributes to CSC properties and aggressive tumor behavior 13. Targeting these markers offers therapeutic potential but requires careful consideration because of their roles in normal stem cell functions.

Chronic inflammation promotes colorectal cell growth through immune system pathway dysregulation. Immune cell infiltration, such as macrophages, releases proinflammatory cytokines (IL-6, IL-17, TNF-α), which enhance tumor proliferation, survival, and invasion. IL-17RA plays a significant role in cancer by mediating the effects of IL-17, a proinflammatory cytokine. When IL-17 binds to IL-17RA, it triggers various intracellular signaling pathways, including JAK2/STAT3, PI3K/Akt, and NF-κB. These pathways promote the proliferation, survival and metastasis of cancer cells. 48, 49 In addition to promoting tumor growth and metastasis, IL-17 signaling has been shown to enhance tumor angiogenesis 30, influence the composition of the tumor-infiltrating leukocyte population 21, 50, 51, and decrease susceptibility to chemotherapy 22 and anti-VEGF therapy 50. Specifically, IL-17 activates STAT3 to promote the transformation of quiescent gastric CSC, drive the progression of cervical cancer, and induce epithelial-mesenchymal transition in lung adenocarcinoma 52, 53. Moreover, the EGF-STAT3 signaling pathway plays a crucial role in promoting and maintaining the stemness of CRC 54. In various cancers, including CRC, both IL-17 and its receptor, IL-17RA, are highly expressed in tumor tissues compared with normal tissues 29, 31, 55. Our previous and current studies found that IL-17 is highly expressed in CRC tumors and is negatively correlated with survival 31. This study demonstrated that IL-17RA overexpression promotes CSC properties and 5-FU resistance. The mechanism by which IL-17RA signaling modulates CSC properties through STAT3 activation suggests that IL-17RA could serve as a potential therapeutic target for patients with late-stage CRC. Recently, a global population-based cohort study showed that patients with psoriasis who were prescribed an IL-17 inhibitor had a decreased risk of colorectal cancer 56. Brodalumab, an IL-17RA antagonist that is the most effective for psoriasis treatment, may be considered in clinical trials as a regimen for CRC treatment 57.

## Supplementary Material

Supplementary figures.

## Figures and Tables

**Table 1:** The demographic and clinicopathological characteristics of patients with CRC.

Variables	Patient Number	%
**Total**	68	100
**Age**		
≥ 65	36	57
< 65	32	43
**Gender**		
Male	44	48
Female	24	52
TNM Stage		
I	6	9
II	28	41
III	25	37
IV	9	13
**Tumor stage**		
T1-T2	9	13
T3-T4	59	87
**Node Status**		
N0	35	51
N1	20	29
N2	13	19
**Distant Metastasis**		
M0	59	87
M1	9	13
**Location**		
Right colon	19	28
Left colon	42	62
Rectal	7	10
**CEA (5 ng/ml)**		
> 5	28	41
≦5	40	59
**CA19-9 (U/ml)**		
> 37	27	40
≦37	39	57
**MSI**		
MSS	63	93
MSI-H	5	7

## References

[1] Sung H, Ferlay J, Siegel RL, Laversanne M, Soerjomataram I, Jemal A (2021). Global Cancer Statistics 2020: GLOBOCAN Estimates of Incidence and Mortality Worldwide for 36 Cancers in 185 Countries. CA: a cancer journal for clinicians.

[2] Siegel RL, Miller KD, Goding Sauer A, Fedewa SA, Butterly LF, Anderson JC (2020). Colorectal cancer statistics, 2020. CA: a cancer journal for clinicians.

[3] Russo M, Crisafulli G, Sogari A, Reilly NM, Arena S, Lamba S (2019). Adaptive mutability of colorectal cancers in response to targeted therapies. Science.

[4] van der Geest LG, Lam-Boer J, Koopman M, Verhoef C, Elferink MA, de Wilt JH (2015). Nationwide trends in incidence, treatment and survival of colorectal cancer patients with synchronous metastases. Clinical & experimental metastasis.

[5] Brabletz T, Jung A, Spaderna S, Hlubek F, Kirchner T (2005). Opinion: migrating cancer stem cells - an integrated concept of malignant tumour progression. Nature reviews Cancer.

[6] Pardal R, Clarke MF, Morrison SJ (2003). Applying the principles of stem-cell biology to cancer. Nature reviews Cancer.

[7] Shimokawa M, Ohta Y, Nishikori S, Matano M, Takano A, Fujii M (2017). Visualization and targeting of LGR5(+) human colon cancer stem cells. Nature.

[8] Rezaee M, Gheytanchi E, Madjd Z, Mehrazma M (2021). Clinicopathological Significance of Tumor Stem Cell Markers ALDH1 and CD133 in Colorectal Carcinoma. Iran J Pathol.

[9] Beier D, Hau P, Proescholdt M, Lohmeier A, Wischhusen J, Oefner PJ (2007). CD133(+) and CD133(-) glioblastoma-derived cancer stem cells show differential growth characteristics and molecular profiles. Cancer Res.

[10] Cao W, Li M, Liu J, Zhang S, Noordam L, Verstegen MMA (2020). LGR5 marks targetable tumor-initiating cells in mouse liver cancer. Nat Commun.

[11] Roudi R, Korourian A, Shariftabrizi A, Madjd Z (2015). Differential Expression of Cancer Stem Cell Markers ALDH1 and CD133 in Various Lung Cancer Subtypes. Cancer Invest.

[12] Roudi R, Barodabi M, Madjd Z, Roviello G, Corona SP, Panahei M (2020). Expression patterns and clinical significance of the potential cancer stem cell markers OCT4 and NANOG in colorectal cancer patients. Mol Cell Oncol.

[13] Elbadawy M, Usui T, Yamawaki H, Sasaki K (2019). Emerging Roles of C-Myc in Cancer Stem Cell-Related Signaling and Resistance to Cancer Chemotherapy: A Potential Therapeutic Target Against Colorectal Cancer. Int J Mol Sci.

[14] Zhu Y, Huang S, Chen S, Chen J, Wang Z, Wang Y (2021). SOX2 promotes chemoresistance, cancer stem cells properties, and epithelial-mesenchymal transition by beta-catenin and Beclin1/autophagy signaling in colorectal cancer. Cell Death Dis.

[15] Gaffen SL (2011). Recent advances in the IL-17 cytokine family. Current opinion in immunology.

[16] Parajuli P, Mittal S (2013). Role of IL-17 in Glioma Progression. Journal of spine & neurosurgery.

[17] Prabhala RH, Pelluru D, Fulciniti M, Prabhala HK, Nanjappa P, Song W (2010). Elevated IL-17 produced by TH17 cells promotes myeloma cell growth and inhibits immune function in multiple myeloma. Blood.

[18] Iwakura Y, Ishigame H, Saijo S, Nakae S (2011). Functional specialization of interleukin-17 family members. Immunity.

[19] Reynolds JM, Angkasekwinai P, Dong C (2010). IL-17 family member cytokines: regulation and function in innate immunity. Cytokine Growth Factor Rev.

[20] Wu L, Chen X, Zhao J, Martin B, Zepp JA, Ko JS (2015). A novel IL-17 signaling pathway controlling keratinocyte proliferation and tumorigenesis via the TRAF4-ERK5 axis. J Exp Med.

[21] He D, Li H, Yusuf N, Elmets CA, Li J, Mountz JD (2010). IL-17 promotes tumor development through the induction of tumor promoting microenvironments at tumor sites and myeloid-derived suppressor cells. J Immunol.

[22] Wang K, Kim MK, Di Caro G, Wong J, Shalapour S, Wan J (2014). Interleukin-17 receptor a signaling in transformed enterocytes promotes early colorectal tumorigenesis. Immunity.

[23] Li Q, Han Y, Fei G, Guo Z, Ren T, Liu Z (2012). IL-17 promoted metastasis of non-small-cell lung cancer cells. Immunology letters.

[24] Hyun YS, Han DS, Lee AR, Eun CS, Youn J, Kim HY (2012). Role of IL-17A in the development of colitis-associated cancer. Carcinogenesis.

[25] Carmi Y, Rinott G, Dotan S, Elkabets M, Rider P, Voronov E (2011). Microenvironment-derived IL-1 and IL-17 interact in the control of lung metastasis. Journal of immunology (Baltimore, Md: 1950).

[26] Sun Y, Pan J, Mao S, Jin J (2014). IL-17/miR-192/IL-17Rs regulatory feedback loop facilitates multiple myeloma progression. PLoS ONE.

[27] Cochaud S, Giustiniani J, Thomas C, Laprevotte E, Garbar C, Savoye AM (2013). IL-17A is produced by breast cancer TILs and promotes chemoresistance and proliferation through ERK1/2. Scientific reports.

[28] Wu Z, He D, Zhao S, Wang H (2019). IL-17A/IL-17RA promotes invasion and activates MMP-2 and MMP-9 expression via p38 MAPK signaling pathway in non-small cell lung cancer. Mol Cell Biochem.

[29] Jiang YX, Li PA, Yang SW, Hao YX, Yu PW (2015). Increased chemokine receptor IL-17RA expression is associated with poor survival in gastric cancer patients. Int J Clin Exp Pathol.

[30] Wang M, Wang L, Ren T, Xu L, Wen Z (2013). IL-17A/IL-17RA interaction promoted metastasis of osteosarcoma cells. Cancer Biol Ther.

[31] Tseng JY, Yang CY, Liang SC, Liu RS, Yang SH, Lin JK (2014). Interleukin-17A modulates circulating tumor cells in tumor draining vein of colorectal cancers and affects metastases. Clinical cancer research: an official journal of the American Association for Cancer Research.

[32] Jiang JK, Lin CH, Chang TA, Lo LC, Lin CP, Lu RH (2024). Decreased interleukin-17RA expression is associated with good prognosis in patients with colorectal cancer and inhibits tumor growth and vascularity in mice. Cancer Med.

[33] Parajuli P, Anand R, Mandalaparty C, Suryadevara R, Sriranga PU, Michelhaugh SK (2016). Preferential expression of functional IL-17R in glioma stem cells: potential role in self-renewal. Oncotarget.

[34] Melcher R, Steinlein C, Feichtinger W, Muller CR, Menzel T, Luhrs H (2000). Spectral karyotyping of the human colon cancer cell lines SW480 and SW620. Cytogenet Cell Genet.

[35] Livak KJ, Schmittgen TD (2001). Analysis of relative gene expression data using real-time quantitative PCR and the 2(-Delta Delta C(T)) Method. Methods.

[36] Florek M, Haase M, Marzesco AM, Freund D, Ehninger G, Huttner WB (2005). Prominin-1/CD133, a neural and hematopoietic stem cell marker, is expressed in adult human differentiated cells and certain types of kidney cancer. Cell Tissue Res.

[37] Shmelkov SV, Butler JM, Hooper AT, Hormigo A, Kushner J, Milde T (2008). CD133 expression is not restricted to stem cells, and both CD133+ and CD133- metastatic colon cancer cells initiate tumors. J Clin Invest.

[38] Bhutada I, Khambati F, Cheng SY, Tiek DM, Duckett D, Lawrence H (2024). CDK7 and CDK9 inhibition interferes with transcription, translation, and stemness, and induces cytotoxicity in GBM irrespective of temozolomide sensitivity. Neuro Oncol.

[39] Razali N M, Yap B W (2011). Power comparisons of Shapiro-Wilk, Kolmogorov-Smirnov, Lilliefors and Anderson-Darling tests. Journal of Statistical Modeling and Analytics.

[40] Clevers H (2011). The cancer stem cell: premises, promises and challenges. Nature medicine.

[41] Dean M, Fojo T, Bates S (2005). Tumour stem cells and drug resistance. Nature reviews Cancer.

[42] Zhao H, Liu Q, Wang S, Dai F, Cheng X, Cheng X (2017). *In vitro* additive antitumor effects of dimethoxycurcumin and 5-fluorouracil in colon cancer cells. Cancer Med.

[43] Slater C, De La Mare JA, Edkins AL (2018). *In vitro* analysis of putative cancer stem cell populations and chemosensitivity in the SW480 and SW620 colon cancer metastasis model. Oncol Lett.

[44] Luo Q, Liu Y, Shi K, Shen X, Yang Y, Liang X (2023). An autonomous activation of interleukin-17 receptor signaling sustains inflammation and promotes disease progression. Immunity.

[45] Moreno-Londono AP, Robles-Flores M (2024). Functional Roles of CD133: More than Stemness Associated Factor Regulated by the Microenvironment. Stem Cell Rev Rep.

[46] Xu L, Lin W, Wen L, Li G (2019). Lgr5 in cancer biology: functional identification of Lgr5 in cancer progression and potential opportunities for novel therapy. Stem Cell Res Ther.

[47] Wei Y, Li Y, Chen Y, Liu P, Huang S, Zhang Y (2022). ALDH1: A potential therapeutic target for cancer stem cells in solid tumors. Front Oncol.

[48] Wu D, Wu P, Huang Q, Liu Y, Ye J, Huang J (2013). Interleukin-17: a promoter in colorectal cancer progression. Clinical & developmental immunology.

[49] De Simone V, Pallone F, Monteleone G, Stolfi C (2013). Role of TH17 cytokines in the control of colorectal cancer. Oncoimmunology.

[50] Chung AS, Wu X, Zhuang G, Ngu H, Kasman I, Zhang J (2013). An interleukin-17-mediated paracrine network promotes tumor resistance to anti-angiogenic therapy. Nature medicine.

[51] Martin-Orozco N, Muranski P, Chung Y, Yang XO, Yamazaki T, Lu S (2009). T helper 17 cells promote cytotoxic T cell activation in tumor immunity. Immunity.

[52] Bai Y, Li H, Lv R (2021). Interleukin-17 activates JAK2/STAT3, PI3K/Akt and nuclear factor-kappaB signaling pathway to promote the tumorigenesis of cervical cancer. Exp Ther Med.

[53] Huang Q, Han J, Fan J, Duan L, Guo M, Lv Z (2016). IL-17 induces EMT via Stat3 in lung adenocarcinoma. Am J Cancer Res.

[54] Cheng CC, Liao PN, Ho AS, Lim KH, Chang J, Su YW (2018). STAT3 exacerbates survival of cancer stem-like tumorspheres in EGFR-positive colorectal cancers: RNAseq analysis and therapeutic screening. J Biomed Sci.

[55] Liu Y, Zhao X, Sun X, Li Y, Wang Z, Jiang J (2015). Expression of IL-17A, E, and F and their receptors in human prostatic cancer: Comparison with benign prostatic hyperplasia. Prostate.

[56] Kridin K, Abdelghaffar M, Mruwat N, Ludwig RJ, Thaci D (2024). Are interleukin 17 and interleukin 23 inhibitors associated with malignancies?-Insights from an international population-based study. J Eur Acad Dermatol Venereol.

[57] Song M, Liang J, Wang L, Li W, Jiang S, Xu S (2023). IL-17A functions and the therapeutic use of IL-17A and IL-17RA targeted antibodies for cancer treatment. Int Immunopharmacol.

