# Supplementary material for: Interleukin-17 receptor A drives cancer stem-like properties in colorectal cancer through STAT3 activation
Source: J Cancer. 2026 Jan 1;17(1):74–85. doi: 10.7150/jca.121654 (PMC12719591; doi:10.7150/jca.121654)
Supplement: Supplementary file 1 — Supplementary figures. [file jcav17p0074s1.pdf]

## Supplementary Material

# Interleukin-17 receptor A drives cancer stem-like properties in colorectal cancer through STAT3 activation

**Jeng-Kai Jiang, Chi-Hung Lin, Chun-Chi Lin, Liang-Chuan Lo, Po-Yen Sung, Zhen-Yu Wen, Chien-Ping Lin, Ting-An Chang, Chih-Yung Yang**

Supplemental Fig 1

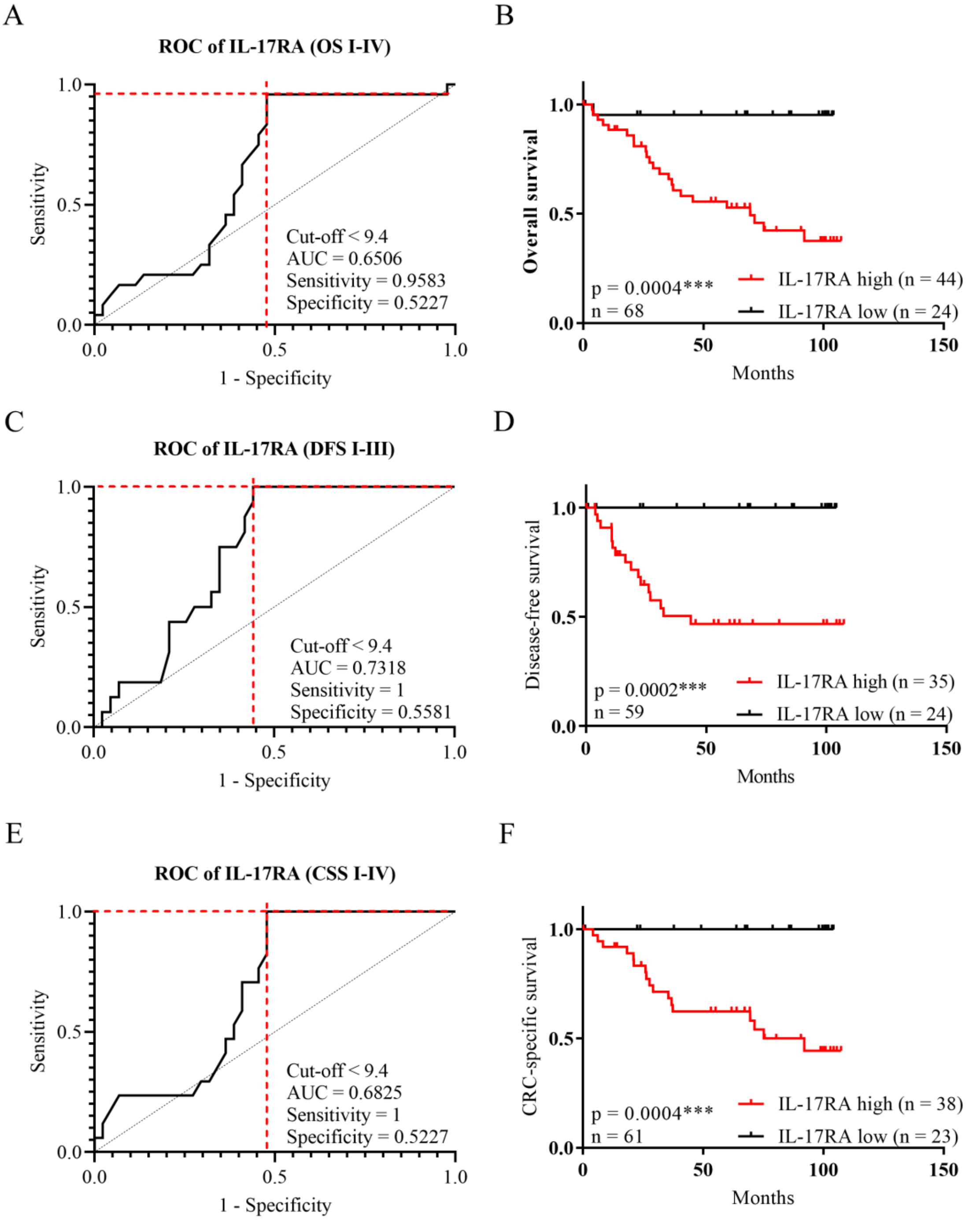


**Supplementary Figure 1** The receiver operating characteristic (ROC) curve and Kaplan–Meier survival curves of survival of patients at all colorectal cancer stages, stratified by interleukin-17 receptor A (IL-17RA) expression. (A, C, E) The ROC curve showing IL-17RA expression as the optimal threshold to use for indicated survival analysis such as Kaplan–Meier overall survival (OS) (B), Disease-free survival (DFS) (D) and cancer-specific survival (CSS) (f) curves for patients with high or low Il-17RA

expression.

Supplemental Fig 2

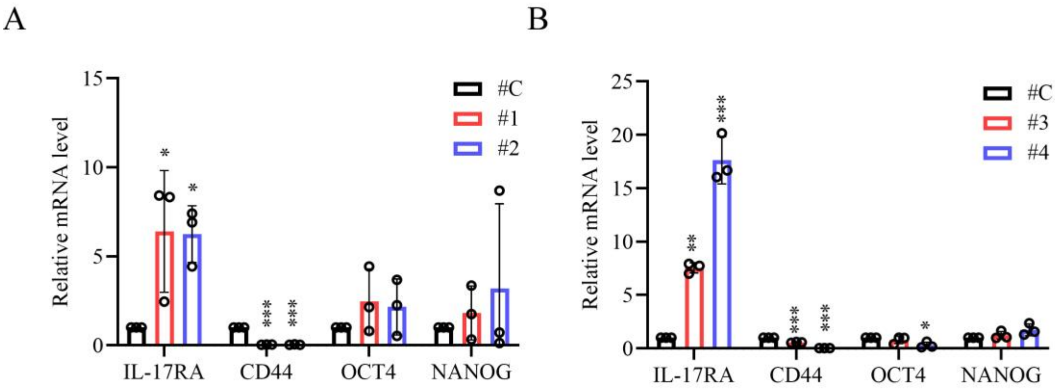


**Supplementary Figure 2** Interleukin-17 receptor A (IL-17RA) overexpression affects the expression of cancer stem cell markers, cluster of differentiation 44 (CD44), Octamer-binding transcription factor (Oct-4) and NF-κB, nuclear factor-κB (Nanog), in SW620 (A) and SW480 (B) cells. mRNA expression of the genes is detected using quantitative RT-PCR. Data are presented as mean ± SD. Statistical significance was determined using one-way ANOVA followed by Dunnett's test. *$p < 0.05$, **$p < 0.01$, ***$p < 0.001$.

Supplemental Fig 3

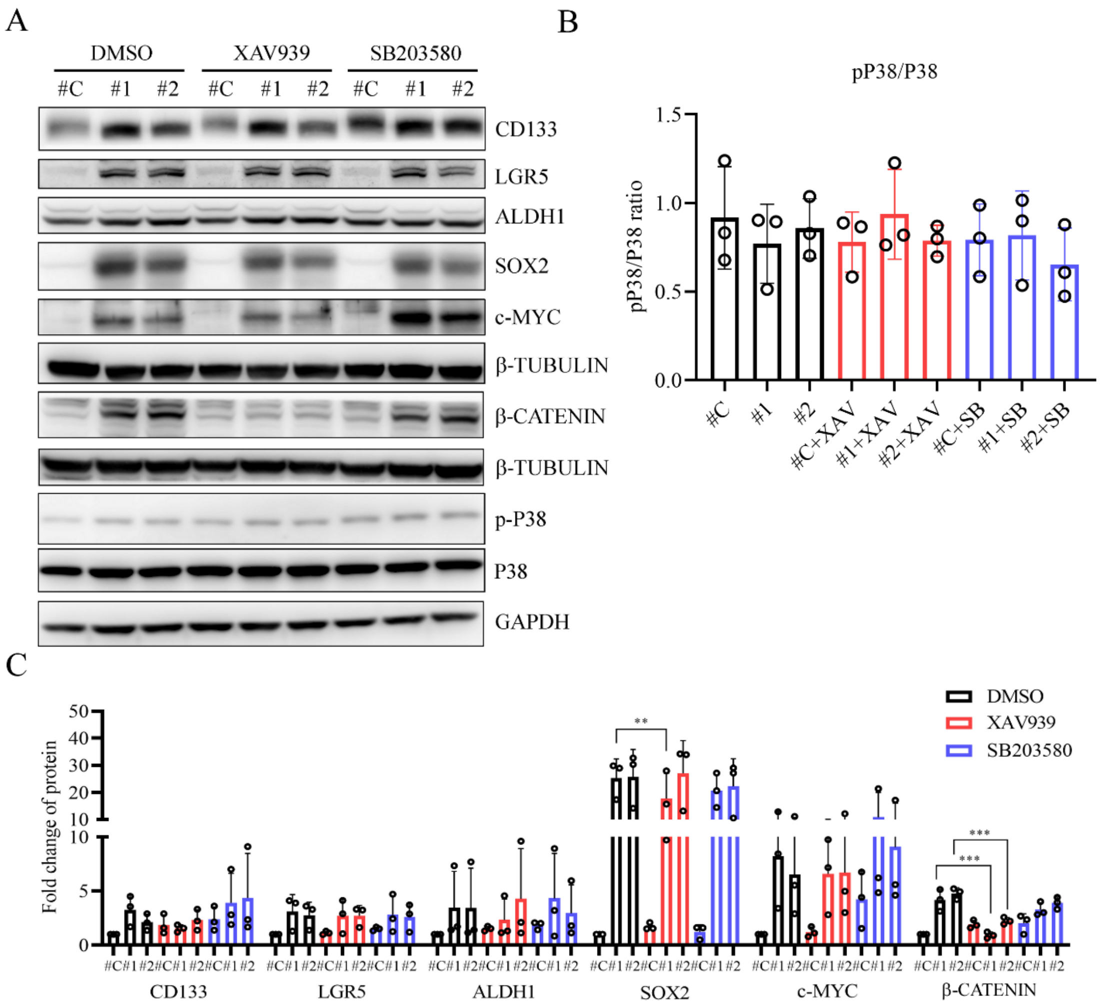


**Supplementary Figure 3** The XAV939 (XAV) and SB203580 (SB) do not affect the expression of stem cell markers in IL-17RA-overexpressing SW620 cells (A). The figure contains protein bands obtained from three separate blots, each with its respective loading control (β-tubulin, β-tubulin, and GAPDH, respectively). Phosphorylated P38 was normalized to total P38 and quantified in (B). XAV939 and SB203580 did not inhibit the indicated CSC markers, which were quantified from three independent experiments, as shown in (C). Data are presented as mean ± SD. Statistical significance was determined using two-way ANOVA followed by Sidak's multiple comparisons test. **p* < 0.05, ***p* < 0.01, ****p* < 0.001.

Supplemental Fig 4

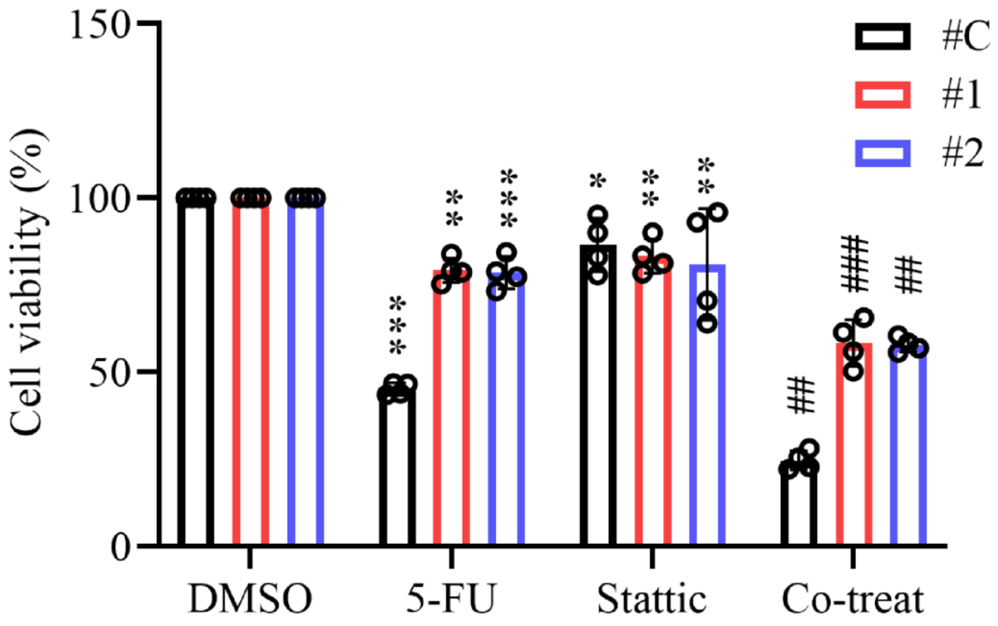


Supplementary Figure 4 Stattic enhances the cytotoxic effect of 5-fluorouracil (5-FU) in interleukin-17 receptor A-overexpressing SW620 cells. Data are presented as mean ± SD. Statistical significance was determined using two-way ANOVA followed by Sidak's multiple comparisons test. *p < 0.05, **p < 0.01, ***p < 0.001 compared to DMSO treatment. #p < 0.05, ###p < 0.001 compared to 5-FU treatment.
